# Three Centuries of Appendicectomy

**DOI:** 10.1007/s00268-022-06874-6

**Published:** 2022-12-29

**Authors:** Philip J. J. Herrod, Alex T. Kwok, Dileep N. Lobo

**Affiliations:** 1 Gastrointestinal Surgery, Nottingham Digestive Diseases Centre and National Institute for Health Research (NIHR) Nottingham Biomedical Research Centre, Nottingham University Hospitals NHS Trust and University of Nottingham, Queen’s Medical Centre, Nottingham, NG7 2UH UK; 2 MRC Versus Arthritis Centre for Musculoskeletal Ageing Research, School of Life Sciences, University of Nottingham, Queen’s Medical Centre, Nottingham, UK

## Abstract

**Background:**

Save for the contribution of Charles McBurney, who described his eponymous point and the appendicectomy incision, the history of appendicectomy is largely unknown among the medical profession. This review traces the history from the first anatomical depiction of the appendix to the development of open appendicectomy and the recent minimally invasive and non-operative methods.

**Methods:**

Historical articles, monographs and books containing anatomical descriptions of the vermiform appendix and reports of appendicitis and its surgical treatment were retrieved after searching the PubMed, Google Scholar and Embase databases from their inception to 31 March 2022.

**Results:**

The first inadvertent appendicectomy was performed during an operation for a groin hernia by Cookesley in 1731, and Mestivier was the first to drain a right iliac fossa abscess, due to appendicitis, in 1757. Krönlein performed the first appendicectomy for acute appendicitis in 1884 but his patient died. The first successful appendicectomy for acute appendicitis leading to patient survival was by Morton in 1887. In 1976, Wirschafter and Kaufman performed an inadvertent colonoscopic appendicectomy and, in 1980, Semm carried out the first laparoscopic appendicectomy. The first appendicectomy via a natural orifice (transgastric) appendicectomy was by Rao and Reddy in 2004.

**Conclusion:**

This historical review charts the development of surgical knowledge concerning the management of appendicitis, from the first anatomical drawings of the appendix and descriptions of appendicitis to the development of surgical and conservative treatments up to the present day. It also corrects some inaccuracies of attribution in previous historical reviews.

## Introduction

The origins and evolution of appendicectomy appear to be shrouded in a degree of mystery, with varying accounts and different surgeons being cited and given credit by various authors [1–5]. The most widely known name is that of Charles Herber McBurney, owing to his description of the point of tenderness on the abdominal wall and the incision for appendicectomy which carry his name [6]. However, appendicectomy predates his work by more than a century.

The aim of this review was to trace the origins of appendicectomy, by reference to the original literature, and to outline an accurate history of this widely practiced operation as well as other aspects of management.

## Methods

The methods employed for this review are described in Table 1.

**Table 1:** Methods and search strategy

Databases searched	PubMed, Google Scholar and Embase
Period of search	From inception to 31 March 2022
Search terms	“vermiform appendix”, “appendicitis”, “typhlitis”, “appendicectomy”, “appendectomy”, “history”, “historical”, “incisions”, “laparoscopic”, “endoscopic”, “NOTES” and “first” in various combinations with the Boolean operators “AND”, “OR” and “NOT”
Language restriction	None
Further searches	The bibliographies of retrieved articles were hand searched for further references
Publication retrieval	Full text articles, monographs and books that described historical aspects of anatomical descriptions of the vermiform appendix, appendicitis and surgery for appendicitis were retrieved from the publishers’ websites as well as https://books.google.com, https://archive.org, https://www.jstor.org and https://www.digitale-sammlungen.de/de, and the British Library Document Supply Service
Translation	Articles in languages other than English were translated into English using Google Translate (https://translate.google.com)

### Anatomical descriptions

**Fig. 1:**
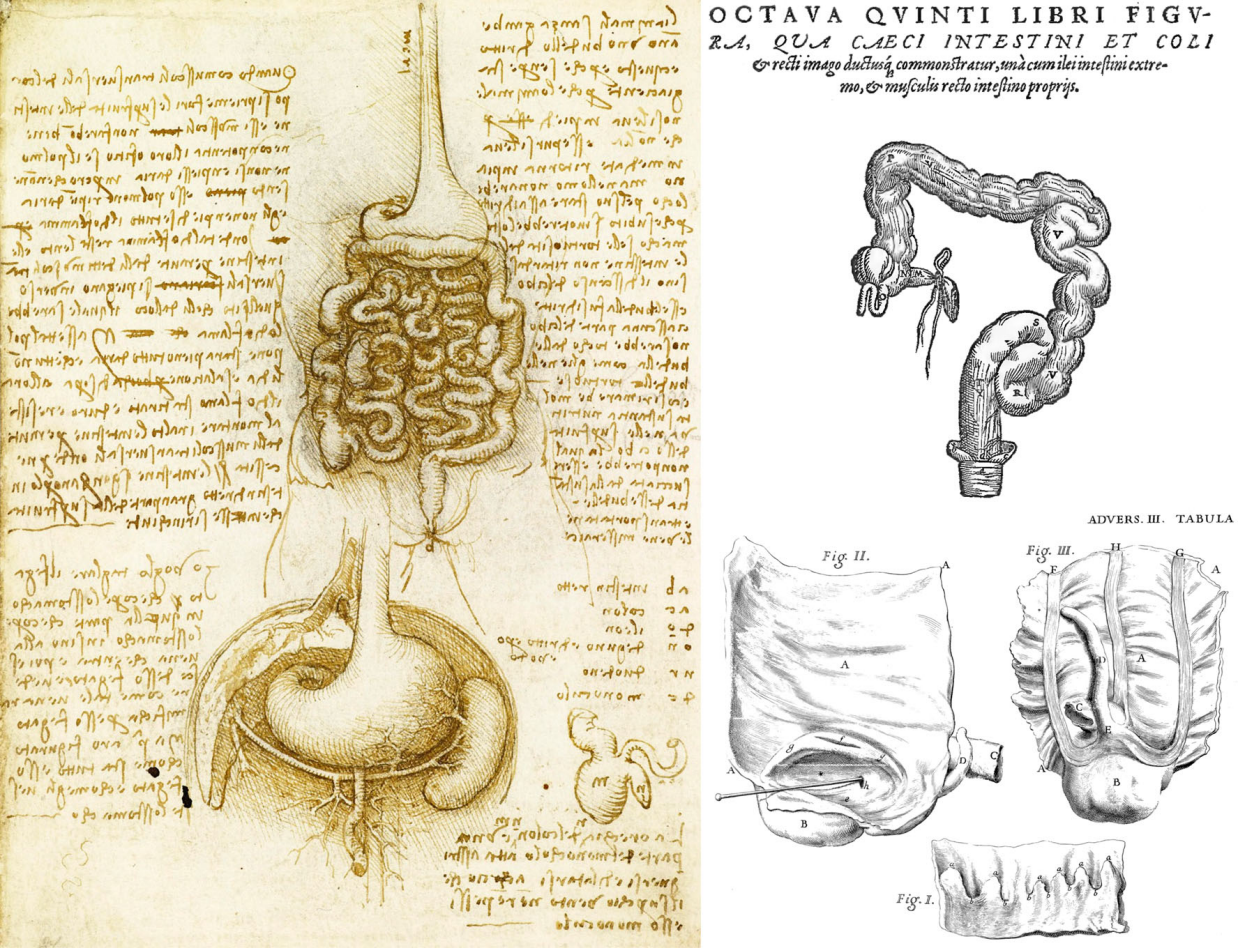
Anatomical drawings of the appendix by Leonardo Da Vinci [7] (left), Andreas Vesalius [9] (upper right) and Giovanni Battista Morgagni [10] (lower right)

The first recorded anatomical drawing of the appendix was that of Leonardo Da Vinci, circa 1508 [7] (Fig. 1) and the first written narrative of the appendix was by Berengarius of Carpi in 1522 [8], who described it as “its shape appeareth strictly compacted, but within it is empty, and is less in breadth than the least finger of the hand, and it is of the length of three inches or thereabouts”.

It was subsequently depicted by the anatomist Andreas Vesalius in De Humani Corporis Fabrica Libri Septem in 1543 [9]. Giovanni Battista Morgagni also produced drawings of the appendix in his 1719 book [10], showing that the appendix had a lumen which was in continuity with the caecum.

### Diagnosis

**Fig. 2:**
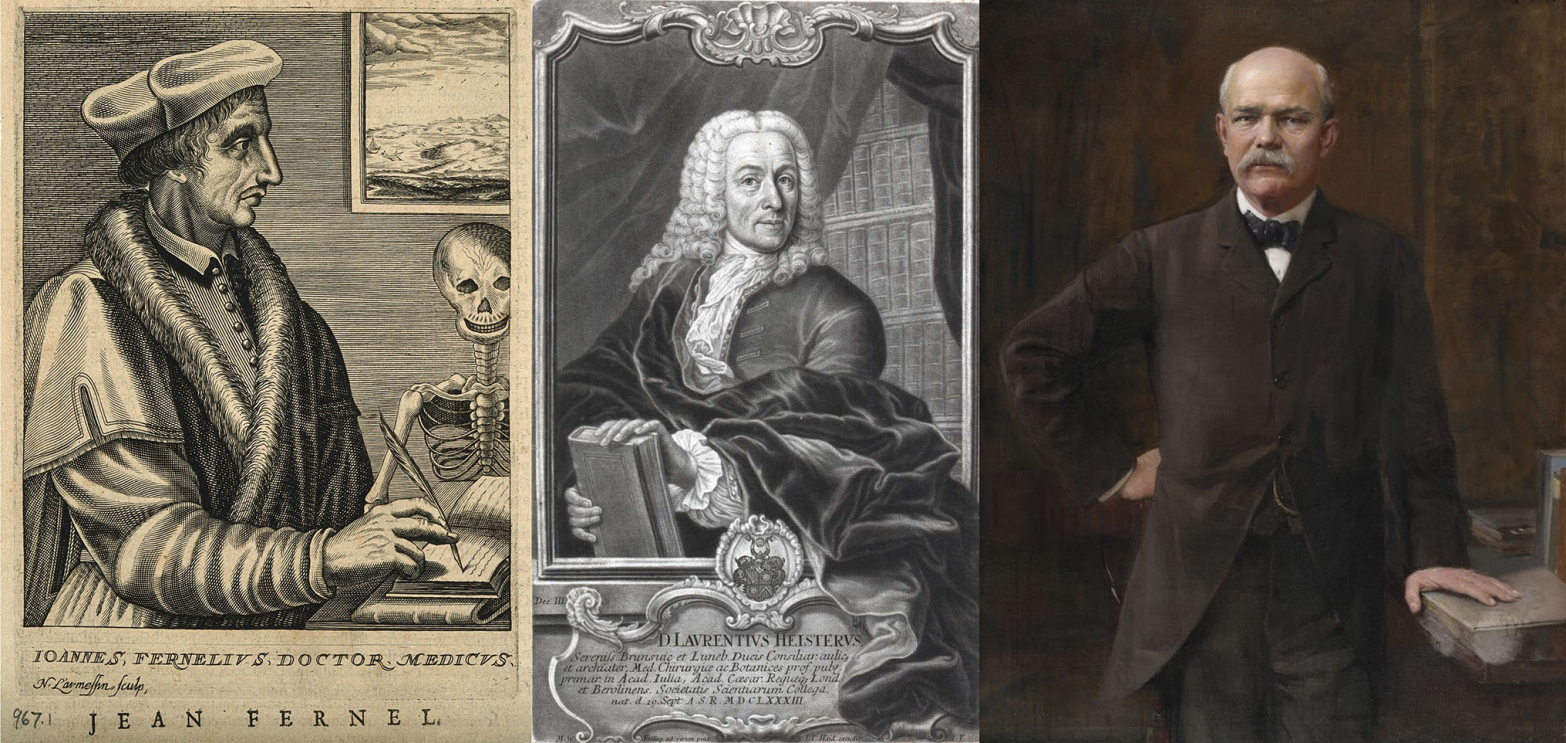
Physicians who described the pathology of appendicitis: Joannis Fernel (left, from the Wellcome Collection), Lorenz Heister (middle, from Wikipedia) and Reginald Herber Fitz (right, portrait by Ignaz Gaugengigl)

The history of the diagnosis of acute appendicitis is confusing, with a variety of descriptions such as “typhilits”, “perityphilitis” and “iliac passion”, until the pathologist Reginald Herber Fitz (Fig. 2), in a review of 257 cases in 1886, popularised the idea that the vermiform appendix was the cause of acute right iliac fossa inflammation and ensuing abscesses [11].

Prior to this, the earliest description of a supposed case of appendicitis was credited to Desiderius Erasmus by Seal [3] quoting Cope [12]. In a letter, written in 1530, Erasmus describes a 3-month illness with abdominal pain, with subsequent development of a hard swelling and an abscess, which was finally incised. However, on closer scrutiny of more recent translations of Erasmus’ correspondence, his symptoms were on the left side of his abdomen [13], making appendicitis unlikely.

Another possible description of acute appendicitis was made in 1554 by Joannis Fernel [14] (Fig. 2), when he described the autopsy finding of a perforation in the caecum of a 7-year-old girl who died following a short history of abdominal pain. However, whether this represented a case of appendicitis is disputed, as the appendix itself was not described [2].

The first definitive account of appendicitis was published by Lorenz Heister (Fig. 2) in 1753, in which he described pathological findings of a perforated appendix with a surrounding abscess at an autopsy performed in 1711 [15].

### Drainage of right iliac fossa abscesses, without appendicectomy

The first published account of an operation to drain an abscess in the right iliac fossa, resulting from disease of the appendix, was performed by Mestivier in 1757 [16]. Unfortunately, the patient died soon after the procedure, and autopsy revealed a perforation of the appendix by a large pin to be the source of the abscess.

**Fig. 3:**
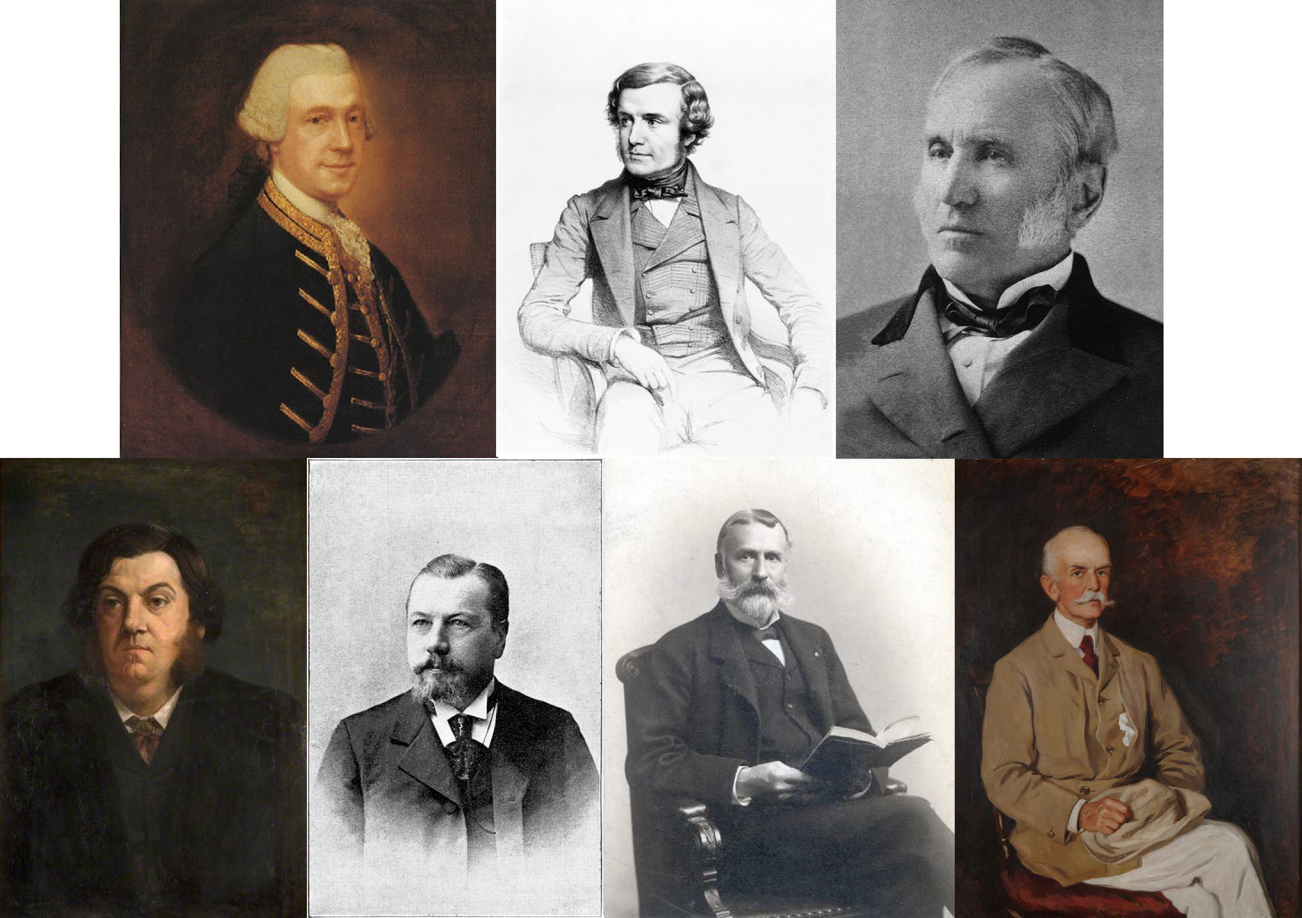
Some surgical pioneers. Top row (from left to right): Claudius Amyand (portrait by Thomas Gainsborough), Henry Hancock (from the Wellcome Collection) and Willard Parker (from Wikipedia). Bottom row (from left to right): Robert Lawson Tait (from the University of Birmingham collection), Rudolf Ulrich Krönlein (from Wikipedia), Thomas George Morton (from PennMed—University of Pennsylvania Image Gallery), Charles Herber McBurney (portrait by Ellen Emmet Rand)

The first publication of a successful operation, with survival of the patient, to drain an appendix abscess in the right iliac fossa, was by Henry Hancock (Fig. 3) in 1848 in a young woman who had gone into premature labour, four days after developing right sided abdominal pain [17]. Fourteen days after the onset of pain, and after she had deteriorated significantly, Hancock made a four-inch incision in her right iliac fossa under chloroform anaesthesia, which caused discharge of “a quantity of excessively offensive turbid serum”. Two weeks later a “small round ball of faecal matter, surrounded by calcareous deposit” was discharged from the wound that had been left open. Hancock concluded that this may have been impacted in the appendix and escaped when it ulcerated.

However, there may have been an operation which predates that of Hancock. A letter by Willard Parker (Fig. 3), published in 1867 [18], describes an operation performed on a local physician in 1843 for an abscess in the right iliac fossa which had developed after several weeks of abdominal pain and fever. Parker describes cutting down and evacuating pus together with a “little concretion, the size of a raisin seed”. The patient recovered and was “in good health” at the time of publication [18].

Robert Lawson Tait (Fig. 3) also described a series of cases of drainage of abscesses associated with typhlitis, the earliest of which was in 1867 [19].

### Incidental appendicectomies during operations for groin herniae

The best-known early report of an operation to remove an appendix is by Claudius Amyand in 1735 (Fig. 3) [20]. He described an 11-year-old boy undergoing an operation for a right groin hernia which had developed an enterocutaneous fistula. Amyand opened the hernial sac and encountered the “appendix cœci” (sic), which had been perforated by a pin. He proceeded to amputate the appendix and ligate its base and left the wound to heal by secondary intention.

A report by a “surgeon apothecary” (forerunner of general practitioners) called William Cookesley, not published until 1742 [21], describes an operation performed on Abraham Pike, a chimney sweep, in 1731 for a strangulated inguinal hernia. Cookesley excised necrotic small bowel and the patient recovered. When the patient died 31 years later, an autopsy in 1763 by John Symons, a surgeon from Exeter and pupil of William Hunter, revealed an intact small bowel, but a missing caecal pole and appendix [22]. Symons sent Hunter the excised bowel who prepared and preserved the specimen which is now in the Hunterian Museum in Glasgow. Thus, it may be that Cookesley carried out the first appendicectomy (albeit inadvertently) during his treatment of a hernia.

### Appendicectomy through abdominal incisions

The first documented appendicectomy through an abdominal incision was performed by Tait (Fig. 3) in 1880 and published in 1890 [19]. He describes operating on a 17-year-old woman, with a three-month history of abdominal pain, who developed a right lilac fossa swelling and whom he believed to have generalised peritonitis. He made a midline incision without finding any pus, but when he made an incision “over the caecum” (which we may assume to be a separate skin incision) he found a large abscess containing a gangrenous appendix which he removed, burying the stump. The patient survived and left hospital a month later.

The first recorded appendicectomy through an abdominal incision for acute appendicitis was by Rudolf Ulrich Krönlein (Fig. 3) in 1884 on a 17-year-old blacksmith who had 3 days of abdominal pain and published in 1886 [23]. Using the antiseptic techniques of Lister, Krönlein performed a lower midline laparotomy and evacuated a large amount of pus from the right iliac fossa. He found a circular pea-sized hole in the middle of the appendix and went on to doubly ligate the base of the appendix followed by resection. Unfortunately, the patient died two days later.

The second record of this operation for acute appendicitis, which this time the patient, a 26-year-old man who had suffered from 3 years of episodic abdominal pain, survived, was by Thomas George Morton (Fig. 3) in 1887 and published in 1888 [24]. The episode leading to surgery had started 3 days previously and was now associated with fever. Morton considered him to be “in the dying stages of general purulent peritonitis”. After an abdominal incision, an abscess cavity was encountered, within which he found the caecum and a diseased appendix. He describes a “phosphatic concretion resembling a cherry stone” lying alongside the appendix having been extruded from a perforating ulcer near its base. The appendix was ligated at its base and excised. The patient made an uneventful recovery and was alive a year later at the time of publication.

Morton also described a second patient who had been suffering from intermittent abdominal pain and vomiting for 4 years in whom in 1886 he drained a large amount of foetid pus from a right iliac fossa abscess [24]. However, neither the appendix nor caecum were identified, and the patient was left with an open wound for 5 months. During a recurrent episode in 1888 her appendix was identified in the abscess cavity, with a perforation at its base, and was ligated and excised. Again, the patient survived.

### Incisions

Prior to the use of midline incisions [23], the classical incision for draining abscesses in the right iliac fossa was that described by Hancock, starting at the anterior superior iliac spine and extending medially, parallel to Poupart’s ligament [17]. McBurney’s (Fig. 3) gridiron incision, first described in 1894 [25], is still widely used for open appendicectomy. Over the subsequent 10 years, further incisions were described, varying between vertical and transverse (Table 2) [17, 23, 25–31].

**Table 2:** The development of abdominal incisions for appendicectomy

Oblique Incisions		Horizontal incisions		Vertical incisions	
Incision	Author	Incision	Author(s)	Incision	Author
Starting at right anterior superior iliac spine extending medially, parallel to inguinal ligament	Hancock 1848 [17]	Rockey-Davis	Rockey 1905 [26] and Davis 1906 [27]	Midline	Krönlein 1884 [23]
Gridiron	McBurney 1894 [25]	Lanz	Lanz 1908 [28]	Paramedian	Battle 1895 [29]
Rutherford Morrison	Rutherford Morrison 1896 [30]	Bikini	Delany and Carnevale 1976 [31]		

### Non-operative management

After a rapid increase in the number of operations for appendicitis in the late nineteenth century, suggestions concerning more conservative delayed management were first voiced by Albert John Ochsner in 1902 [32]. In his Handbook of Appendicitis, he advocated delayed management if a trained surgeon with appropriate assistants or an appropriate environment was not immediately available, or if the patient was too unwell to “bear the shock of an operation”. In this pre-antibiotic era Ochsner advocated the provision of morphine and gut rest as management in some cases.

Delayed treatment was refined by James Sherren, with the resultant protocol being named the Ochsner-Sherren treatment [33]. This protocol called for delayed appendicectomy in patients, without generalised peritonitis, presenting with greater than 48 h duration of symptoms. Patients were admitted for bedrest and gut rest, but in this modification no morphine was provided. Prior to this, in 1889, D’arcy Power recognised that “simple appendicitis” may resolve spontaneously when he wrote, “It may be accepted as an axiom that a case of appendicitis which has been properly diagnosed and well treated should recover, for medical treatment will be adopted in the simple cases, and the surgeon will be summoned as soon as the inflammation ceases to run a straight forward course” [34].

### Auto-appendicectomy

The most widely known case of a surgeon removing his own appendix was that of Leonid Ivanovich Rogozov, a Russian surgeon who was the only medical professional on an Antarctic expedition in 1961 [35]. After two days of no improvement with antibiotics, he performed his own appendicectomy under local anaesthesia with the aid of a mirror and three non-medically trained assistants.

It seems, however, that this landmark procedure had been attempted at least 50 years previously (reported in 1912) by an American surgeon Bertram F. Alden, who started his own appendicectomy while under spinal anaesthesia [36]. However, when one of his assistants threatened to leave the theatre unless he stopped, he allowed that assistant to complete the operation [36]. A complete auto-appendicectomy was performed in 1921 by another American surgeon Evan O’Neill Kane [37]. With the aid of three surgically trained assistants, Kane operated, in his own case of uncomplicated acute appendicitis, using only morphine preoperatively and local anaesthesia to the abdominal wall. He made a 3.5-inch-long oblique incision at one inch below the McBurney’s point, which is believed to be longer than the incision he would have normally made because of the uncertainty of operating on himself. Kane also used silk ligature to bury the stump of the appendix, which he claimed to be a method of his own. The pain remained bearable throughout the operation and he made a smooth recovery. He, therefore, advocated the use of local over general anaesthesia in appropriate cases. Kane believed the success of his auto-appendicectomy could be generalised to a wider context—“I wish to emphasize my statement that any surgeons, if not obese, can, with perfect ease and even comfort, self-operate in cases such as mine.” [36].

### Laparoscopic appendicectomy

Stimulated by developments in gynaecological diagnostic laparoscopy, the first laparoscopic appendicectomy was performed by Kurt Semm, a gynaecologist, on 13th September 1980 [38]. His original description involved a 4-port technique, where the appendix was displayed by tying a Roeder knot about its tip, and then the mesoappendix ligated by an extracorporeally thrown knot. Following this, the mesoappendix was cut from the appendix and two Roeder loops applied to the base. The base was then divided and the stump invaginated using both a laparoscopically applied purse-string suture and a subsequent Z-stitch.

### Natural orifice endoscopic surgery

Wirtschafter and Kaufman from California reported the inadvertent endoscopic removal of an inverted appendix in 1976 [39]. The patient underwent a colonoscopy having had a filling defect in her caecum identified on a barium enema. During the colonoscopy, what was thought to be a caecal polyp was removed with a hot snare. The histology report showed the tissue to be “consistent with appendix, showing marked autolysis”. The patient developed right iliac fossa pain three days after the procedure, but was treated with antibiotics and made a full recovery.

The wide adoption of laparoscopic appendicectomy led to interest in peroral transgastric appendicectomy, the first report of which was made at the Annual Congress of the Society of Gastrointestinal Endoscopy of India by Reddy and Rao in 2004 and presented again in 2016 [40]. The first trans-vaginal appendicectomy was reported by Chinnusamy Palanivelu and colleagues in 2008 [41]. There has also been a report of an intentional colonoscopic appendicectomy by Tao Chen and colleagues [42], for a sessile serrated caecal polyp involving appendicular orifice rather than appendicitis, the defect being closed with endoscopic clips.

### Antibiotic therapy

Although appendicectomy had been the mainstay of treatment for appendicitis since the late nineteenth century, it was not until the introduction of antibiotics that some surgeons began to consider non-operative management not only for the treatment of appendicular abscesses, but also for acute appendicitis. In 1956, Eric Coldrey promoted the practice of conservative management for patients with greater than 24 h of symptoms [43]. His regimen consisted of free intake of water by mouth, and six-hourly injections of 250,000 units of penicillin and 0.5 g streptomycin. He recommended chloramphenicol, chlortetracycline, tetracycline, or sulphadimidine for “severe” cases. This strategy remained controversial, with the first randomised clinical trial of antibiotic therapy *versus* appendicectomy not being published until 1995 [44]. The most recent systematic review of 8 randomised clinical trials comparing antibiotic treatment with antibiotics for uncomplicated appendicitis demonstrated the former to be safe [45]. However, 38% of patients randomised to antibiotic treatment required an appendicectomy and had a sixfold greater readmission rate by 1 year [45]. In addition, a randomised clinical trial has shown that a placebo is as effective as antibiotics for the treatment of uncomplicated acute appendicitis [46].

## Conclusion

This review has charted the descriptions and treatments of appendicitis from the first anatomical drawings of the appendix to the recognition of appendicitis and development of appendicectomy, as well as novel forms of management. It has also attempted to correct some of the inaccuracies of attribution in previous reviews and the timeline is summarised in Table 3.

**Table 3:** A timeline of the history of the appendicectomy

1508	First recorded anatomical drawing of the appendix (da Vinci)
1522	First description of the appendix in writing (Berengarius of Carpi)
1711	First definitive description of postmortem findings of appendicitis (Heister)
1731	First inadvertent appendicectomy during an operation on a groin hernia (Cookesley, published 1742)
1735	First published report of appendicectomy during an operation on a groin hernia (Amyand)
1757	First operation draining an abscess in the right iliac fossa from appendicitis (Mestivier)—patient died
1843	First operation to drain an abscess in the right iliac fossa leading to patient survival (Parker, published 1856)
1848	First publication of a successful operation to drain an abscess in the right iliac fossa (Hancock)
1880	First appendicectomy through an abdominal incision (Tait)—patient survived
1884	First appendicectomy for acute appendicitis (Krönlein)—patient died
1886	Publication of a pathological case series of 257 cases of appendicitis- leading to widespread acceptance that the appendix was the primary cause of acute septic right iliac fossa disease (Fitz)
1887	First appendicectomy for acute appendicitis leading to patient survival (Morton)
1894	Publication of gridiron incision (McBurney)
1912	First attempted auto-appendicectomy (Alden)
1921	First completed auto-appendicectomy (Kane)
1956	First description of primary antibiotic treatment of appendicitis in a series of patients (Coldrey)
1976	First inadvertent colonoscopic appendicectomy (Wirtschafter and Kaufman)
1980	First laparoscopic appendicectomy (Semm)
2004	First intentional natural orifice (per oral transgastric) appendicectomy (Reddy and Rao)
2008	First transvaginal appendicectomy (Palanivelu and colleagues)

## Declarations

### Copyright statement

All images that accompany the article are more than 75 years old, and hence outwith copyright rules. All sources have been attributed.

### Data sharing

There are no original data to share. Full texts of works cited in this review will be available for sharing upon reasonable request (Dileep.Lobo@nottingham.ac.uk).

### Provenance and peer review

Not commissioned, externally peer-reviewed.

### Protocol registration

As this was a narrative review, protocol registration was not necessary.
